# Disclosure, transparency, and accountability: a qualitative survey of public sector pharmaceutical committee conflict of interest policies in the World Health Organization South-East Asia Region

**DOI:** 10.1186/s12992-022-00822-8

**Published:** 2022-03-18

**Authors:** Quinn Grundy, Lisa Parker, Anna Wong, Terence Fusire, Deirdre Dimancesco, Klara Tisocki, Helena Walkowiak, Taryn Vian, Jillian Kohler

**Affiliations:** 1 University of Toronto, Suite 130, 155 College Street, Toronto, M5T 1P8 Canada; 2 The University of Sydney, Sydney, Australia; 3 World Health Organization, South East Asia Region Office, New Delhi, India; 4 World Health Organization, Geneva, Switzerland; 5 USAID Medicines, Technologies, and Pharmaceutical Services (MTaPS) Program, Management Sciences for Health, Arlington, USA; 6 University of San Francisco, San Francisco, USA

**Keywords:** Conflict of interest, Pharmaceutical policy, World Health Organization, Southeast Asia, Health policy, Pharmaceutical industry, Essential medicines, Access to medicines

## Abstract

**Background:**

Weak governance over public sector pharmaceutical policy and practice limits access to essential medicines, inflates pharmaceutical prices, and wastes scarce health system resources. Pharmaceutical systems are technically complex and involve extensive interactions between the private and public sectors. For members of public sector pharmaceutical committees, relationships with the private sector can result in conflicts of interest, which may introduce commercial biases into decision-making, potentially compromising public health objectives and health system sustainability. We conducted a descriptive, qualitative study of conflict of interest policies and practices in the public pharmaceutical sector in ten countries in the World Health Organization (WHO) South-East Asia Region (SEAR) (Bangladesh, Bhutan, India, Indonesia, Maldives, Myanmar, Nepal, Sri Lanka, Thailand, and Timor-Leste) between September 2020 and March 2021.

**Results:**

We identified 45 policy and regulatory documents and triangulated documentary data with 21 expert interviews. Key informants articulated very different governance priorities and conflict of interest concerns depending on the features of their country’s pharmaceutical industry, market size, and national economic objectives related to the domestic pharmaceutical industry. Public sector pharmaceutical policies and regulations consistently contained provisions for pharmaceutical committee members to disclose relevant interests, but contained little detail about what should be declared, when, and how often, nor whether disclosures are evaluated and by whom. Processes for preventing or managing conflicts of interest were less well developed than those for disclosure except for a few key procurement processes. Where processes for managing conflicts of interest were specified, the dominant strategy was to recuse committee members with a conflict of interest from relevant work. Policies rarely specified that committee members should divest or otherwise be free from conflicts of interest.

**Conclusions:**

Robust processes for conflict of interest prevention and management could ensure the integrity of decision-making and build public trust in pharmaceutical processes to achieve public health objectives. Upstream approaches including supportive legislative frameworks, the creation of oversight bodies, and strengthening regulatory institutions can also contribute to building cultures of transparency, accountability, and trust.

**Supplementary Information:**

The online version contains supplementary material available at 10.1186/s12992-022-00822-8.

## Background

Access to safe, effective, affordable, and quality-assured medicines is vital to population health. Public pharmaceutical committees and agencies are tasked with making decisions about pharmaceutical products on the public’s behalf, including approving products for market, managing the procurement and reimbursement of pharmaceuticals, and establishing essential product lists, all with serious implications for public health and stewardship of public resources [1]. Pharmaceutical systems are technically complex, from research and development through to service delivery, and involve extensive interactions between the private sector and the public sector. Without sufficient controls, private sector interests can influence what products are registered or selected for reimbursement or procurement, the prices of health products, and how health products are used in ways that further commercial, but not necessarily public health interests [1, 2].

Public sector pharmaceutical committees include technical and clinical experts. Often, individuals with expertise in research or clinical fields are sought by industry to serve as consultants, advisors, investigators, and as influential ‘key opinion leaders’ [3]. Globally, financial relationships between physicians, researchers, and the pharmaceutical industry are common and extend into sponsorship of medical education and research [4]. When members of public sector pharmaceutical committees, or the external experts that advise them, have employment, advisory, consulting, or familial relationships with private sector entities, their obligations are in competition, resulting in a conflict of interest. A conflict of interest is defined as a situation in which the existence of secondary interests or obligations risks compromising (or appearing to compromise) an individual’s primary obligation to make decisions in the public’s interest and on the basis of the best available evidence [5]. Because conflicts of interest are a situation, rather than an act, they do not in themselves constitute a breach of duty or trust [1]. Conflicts of interest differ from corruption, though they may serve as a precursor. Corruption is defined by outcome and intent as “the abuse of entrusted power for private gain” [6]. If conflicts of interest are not addressed, secondary interests can influence public committee decision making in ways that reduce access, inflate prices, or increase inappropriate and unsafe use of essential health products, wasting scarce health system resources [6]. This can in turn undermine public trust in policy processes and the products themselves, and threaten the sustainability of health systems [7].

Globally, the main strategy for addressing conflicts of interest in health-related institutions is disclosure [8, 9]. Other prevention and management strategies are possible (see Table 1) but implementation and transparency around these processes is underdeveloped globally [9]. For example, results from the use of the World Health Organization’s (WHO) pharmaceutical public sector transparency assessment tool over 8 years showed that key pharmaceutical sector committees, such as those responsible for medicines selection, often do not use clear criteria to recruit their members, and face issues in executing their conflict of interest policies [10].

**Table 1:** Preventing and managing conflicts of interest

Proposed frameworks for addressing conflicts of interest centre on policy mechanisms and cultural change to encourage ethical conduct and voluntary compliance. Examples of specific interventions include [1, 11]: • **Policies that define a relevant conflict of interest** in the specific decision-making context and why it matters; • **Disclosure mechanisms** that specify what, when, and how interests should be disclosed, whether and how they are verified, how a conflict of interest is evaluated and the subsequent response (if any); • **Committee selection processes** that account for conflicts of interest, including how members are identified and their qualifications; • **Practical, system-level measures** to prevent conflicts of interest; • **Specific management strategies** to reduce the likelihood that conflicts of interest will pose a risk to the individual’s primary obligation; • **Transparency mechanisms** that make disclosures, policies, management strategies, and decision-making publicly available and accessible; and • **Oversight mechanisms** for monitoring, enforcement, and grievances.	

In 2012, WHO held a global meeting with countries participating in the Good Governance for Medicines Program, which was founded in 2004 to contribute to health systems strengthening and prevent vulnerability to corruption through good governance [12]. Participating countries undertook a three-phase process including a national transparency assessment, development of a national good governance for medicines framework, and implementation of national initiatives to promote good governance for medicines [12]. At the 2012 meeting, participants recommended support to countries including guidance for managing conflicts of interest [12]. As a baseline for such guidance, WHO sought to understand how countries currently manage relationships with pharmaceutical companies and conflicts of interest for public sector pharmaceutical committees and agencies.

The current study aimed to address this knowledge gap. We sought to identify what policies are in place to manage conflict of interest for members of public pharmaceutical committees and agencies, explore how policies are applied, and identify examples of good practices, policy gaps, and challenges. We conducted a policy review of WHO SEAR countries, which includes 11 countries of varying population size, income level, and pharmaceutical industry. Given the diversity of SEAR countries, the purpose of this project was to understand the nature and range of conflict of interest policy development in the region with the aim of informing similar initiatives in other countries and regions. Our results are intended to guide the development of practical documents to support dialogue and capacity building initiatives to improve policies and their implementation on the management of conflict of interest in other countries and regions.

### How conflicts of interest impact access to medicines

The public relies on the national regulatory authority to grant market authorization to only those pharmaceuticals with evidence for effectiveness, safety, and assured quality. If any of the committee members responsible for recommending or granting market authorization have financial interests in the suppliers of the product under consideration, then the resulting conflict of interest poses a risk that decisions could favour company interests rather than public health [1]. For example, analysis of voting patterns by members of the United States Food and Drug Administration Center for Drug Evaluation and Research (CDER) advisory committees found that committee members were more likely to vote in ways that favoured a manufacturer when they had financial relationships exclusively with that manufacturer [13].

Similarly, efforts to promote the appropriate and cost-effective use of medical products such as national formularies or essential product lists should be guided by public health interests. The existence of conflicts of interest may undermine clinician and public confidence in how these tools were developed; this in turn can affect prescribing and medication adherence. For example, a qualitative study of stakeholders’ view of Indonesia’s National Formulary found that in theory, stakeholders supported using a national formulary to guide prescribing. However, in practice, stakeholders, particularly physicians, reported low confidence in the formulary due to lack of transparency around the evidence base and process for decision-making and perception of pharmaceutical industry interference [14]. The lack of confidence led to poor formulary adherence, which was further exacerbated by physicians’ conflicts of interest with the pharmaceutical industry [14].

Lack of transparency around the existence and management of conflicts of interest may undermine the legitimacy of public sector decision-making processes and harm public trust. For example, Teerawattananon and Tritasavit characterized the public’s perception of medicine price negotiation in Thailand (which is a precursor to decisions about reimbursement under universal health coverage) as “a mysterious and endless process that industry uses to lobby decision-makers to introduce new technologies” [15]. To enhance public trust in the process, they suggest several transparency measures including requiring declaration of all conflicts of interest and public documentation of the decision-making process including timelines, methodologies, and evidence sources [15].

There is some evidence to suggest that conflicts of interest may also result in less cost-effective resource use. For example, researchers in Thailand found that in terms of health expenditure per member, the Civil Servant Medical Benefit Scheme spent four times that of the Universal Coverage Scheme during 2012–2015 [16]. This was attributed in part to key differences in terms of the governance, though both schemes are tax-financed [16]. While both schemes referenced the National List of Essential Medicines, the Civil Servant Medical Benefit Scheme relied on the expert opinion of Technical Advisory Committee members and had no process for managing conflicts of interest in their advisory role, while the Universal Coverage Scheme was directly informed by the evidence-based Health Intervention and Technology Assessment Program and implemented procedures to identify, prevent, and manage conflicts of interest [16].

Thus, understanding how countries currently regulate and manage conflicts of interest for public sector pharmaceutical committees and agencies can help assist countries in introducing, improving, and implementing conflict of interest policy. Ultimately, robust conflict of interest policies can enhance transparency and accountability and help mitigate undue influence on decision making in pharmaceutical systems.

## Methods

We conducted a descriptive, qualitative study triangulating two data sources: 1) published conflict of interest policies for public pharmaceutical committees and agencies; and 2) key informant interviews with members of the public pharmaceutical sector. This study focused on all 11 countries in the WHO SEAR and included: Bangladesh, Bhutan, Democratic People’s Republic of Korea, India, Indonesia, Maldives, Myanmar, Nepal, Sri Lanka, Thailand, and Timor-Leste. The study was designed in collaboration with WHO SEAR Office staff and was approved by the University of Toronto Research Ethics Board (#39960). We report these methods according to the COREQ guidelines [17] (Supplementary File 1).

This research employs a critical policy studies methodology, which focuses on the ways that interests, values, and normative assumptions shape and inform how policies are decided and implemented [18]. The goal of a critical policy study is to enhance practical knowledge through understandings of the institutional, cultural, historical, and political contexts in which policy practices occur, and in doing so, to advance public health, equity, and social justice [18].

Beginning September 30th 2020, we identified specific pharmaceutical committees in each country operating at the national level by reviewing recent WHO access to medicines regional reports [19, 20] and conducting online searches. We included pharmaceutical committees at the national level with the following mandates:

- Regulatory authority including committees responsible for making decisions on marketing authorization of medicines;
- Medicines selection including committees responsible for making decisions on the composition of the national essential medicines list or national formulary and committees responsible for conducting health technology assessment to inform medicine selection or procurement in the public health sector;
- Pricing including committees responsible for negotiating, fixing or otherwise controlling medicine prices; and,
- Public procurement including committees responsible for developing specifications or tenders or making final contract decisions/awarding tenders for medicines purchased with public funds.

### Document sampling and data extraction

Between September 30th to October 30th 2020, for each of the identified public sector pharmaceutical committees, we searched Ministry of Health and government websites and conducted structured Google searches to identify documents and articles pertaining to governance and/or conflict of interest. We included policy documents written in English or that could be adequately translated using Google Translate, including policies, procedures, guidelines, relevant laws, ministerial decrees, or regulations that explicitly pertained to committee terms of reference, membership and selection procedures, and any conflict of interest provisions. We also included secondary sources including peer-reviewed journal articles and technical reports or handbooks published by government or prominent non-governmental organizations (NGO) that described the above. During each key informant interview, we sought to validate the sample of documents by specifically asking key informants whether, to their knowledge, the sampled policies were currently implemented, and if additional policies existed. In some cases, key informants described or read aloud policies (e.g., a particular disclosure form or process) during the interview that were not publicly available or were not available in English. We also asked the WHO SEAR Office staff to review the list of sampled policies found through internet searches to identify policies, or categories of policies, we may have missed.

In Excel, we created a structured, open-ended data collection form and extracted data related to each committee’s purpose, functions, membership composition, membership selection processes, the nature of expert involvement, the definition of conflict of interest, and information about any conflict of interest provisions.

### Key informant recruitment and interviews

We purposively recruited individuals who had direct knowledge of and/or experience with policies, procedures and practices for managing conflict of interest of committee members and expert advisors in each country in our sampling frame. We identified prospective key informants through invitations to Ministries of Health, policy and literature searches, our professional networks, and snowball sampling. Prospective participants included civil servant staff at relevant national agencies responsible for implementing code of conduct policies and higher-level supervisors as time and their interest permitted; chairs or secretariat members of public pharmaceutical sector committees that comprised our sampling frame; and in-country experts in pharmaceutical governance and conflicts of interest.

QG and LP conducted all recruitment and the interviews. QG is a PhD-prepared registered nurse with expertise in qualitative methods, health policy, and conflicts of interest. LP is a PhD-prepared physician with expertise in qualitative methods, bioethics, and health policy. Both are English-speaking, white women. QG and LP jointly sent email invitations for an individual interview to prospective informants and scheduled interviews with interested persons at a mutually convenient date and time; the choice of interviewer was largely determined by time zone compatibility. All participants provided informed, written consent to participate and for audio-recording of the interview.

QG and LP conducted semi-structured interviews via telephone or videoconference, guided by an open-ended interview guide (Appendix 1). The interviewer introduced herself as a researcher interested in exploring how conflicts of interest are identified and managed in the context of decision-making for pharmaceutical policy and practice in the participant’s country. Interviews were tailored to informants based on preliminary analysis of relevant documents and literature and the informant’s professional role. Interviews focused on the perceived need for conflict of interest policies, examples of current practices and procedures for managing conflicts of interest, and discussion of perceived gaps, challenges, and enablers. Interviews were recorded, professionally transcribed, and deidentified. Field notes were written after each interview to capture details about the context and nature of the interview such as interview modality (e.g. telephone, Zoom), audio quality, and to document emerging lines of inquiry.

### Data analysis

Sampled documents, secondary sources, interview transcripts, and field notes constituted the text for analysis, with the documents serving as the primary data source. For each country, we first wrote a descriptive overview summary, drawing from the literature, sampled policies, and interview transcripts, outlining details about the key committees of interest, their purpose and functions, committee membership and selection processes, conflict of interest policy provisions, and processes for implementing the policies. We drew on the secondary literature sources to generate summaries that described data on country income groupings, maturity of the regulatory and health systems, and pharmaceutical markets to provide context for the narrative. Then, using established frameworks for identifying, preventing, disclosing, and managing conflicts of interest (see Table 1) [1, 11], the authors QG and LP generated a set of descriptive categories and developed a cross-country descriptive analysis by coding the text of sampled policies, the interview transcripts, and overview memos using these categories and then writing descriptive memos within each category. Descriptive categories included principles, committee selection processes, nature and definition of conflict of interest, disclosure, management strategies, prevention, consequences and impact of conflicts of interest, transparency, and policy priorities. Where possible, we tabulated findings according to country or pharmaceutical regulatory process to highlight the prevalence of policy provisions, good practices, and policy and practice gaps. We then conducted a secondary, narrative analysis by examining concrete stories shared by key informants to explore further why practice diverged from policy recommendations; factors that enable or constrain effective policy implementation; recommendations and needs for improvement; and examples of good practices and lessons learned. Within each interview, we identified concrete narratives – stories with a beginning, middle, and end – and analysed these by writing memos that described what happened, who was involved, core concerns, and how concerns were resolved. We drew on this narrative analysis to generate examples of managing conflict of interest.

### Reflexivity

This study was initiated by staff at the WHO SEAR Office and headquarters as part of ongoing initiatives to strengthen governance processes that impact the availability and accessibility of essential medicines. The study was co-designed by WHO staff and experts on conflicts of interest, corruption, and pharmaceutical governance located in the United States and Canada. Thus, the perspectives we bring to this project are shaped by experiences working on access to medicines in the WHO SEAR, with the WHO Good Governance for Medicines Program, and experiences with conflict of interest policy development in high-income countries. While this study aimed to address an important gap in the literature around the existence and nature of processes for addressing conflicts of interest across a range of lower- and middle-income countries, the composition of this team and the perspectives we bring raise the question of whether and how frameworks (e.g., the IOM framework) developed predominantly in high-income, English-speaking countries with a focus on the medical profession are relevant and/or appropriate in the context of the WHO SEAR.

Though the team benefited from the expertise and networks of WHO SEAR Office technical staff, gaps remained in the team’s knowledge of policy implementation on the ground and ability to search for and read policies in Bangla, Nepali, Thai, and Portuguese. To address these gaps and to seek a range of perspectives on the phenomenon, we elected to conduct key informant interviews with 2–3 individuals per country to gain frontline perspectives on policy implementation, including the existence of policies that were not publicly available in English. Many of the key informants worked within Ministries of Health, the civil service, academia, or independent research units, had conducted research and published on pharmaceutical policy processes, and were recognized experts in their countries. Thus, during interviews and in an effort to challenge our own preconceptions, we asked informants to share these experiences and solicited their views on their understandings of, the need for, approaches to, and relative priority afforded to measures to address conflicts of interest.

## Results

**Fig. 1:**
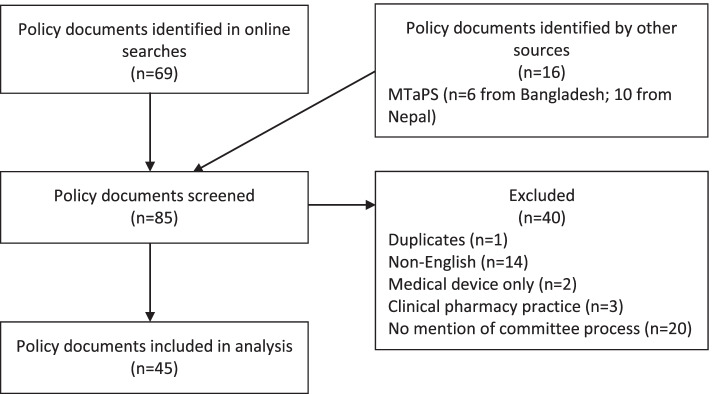
Sampling flow diagram for publicly available policies

We identified 85 publicly available documents across 10 countries. We did not identify any publicly available documents for the Democratic People’s Republic of Korea. Figure 1 outlines the screening process. We included 45 documents for analysis which described the purpose and functions of 41 public sector pharmaceutical committees from 10 countries (see Appendix 2).

We emailed interview invitations to 55 people from the 11 WHO SEAR countries and interviewed 21 individuals from 8 countries during 15 interviews between January and March 2021. Due to the military coup in Myanmar on February 1st, 2021, we were unable to recruit any key informants from this country. We were unable to contact anyone in the Democratic People’s Republic of Korea due to rejection of our emails by the internet server. We were also unable to recruit any informants from Bangladesh, as prospective participants did not respond or declined to participate. Other prospective participants either did not respond or were unable to participate, frequently citing the workload related to the COVID-19 pandemic. Interviews lasted between 30 and 68 min (mean = 45 min). Participants represented a range of disciplines including pharmacy, nursing, health economics, social science, and law and worked in the public sector (including Ministries of Health, national medicines regulatory agencies, civil services), academia and independent research organizations, and the health system.

The 10 sampled countries represented a diverse range of lower- and middle-income countries in terms of size, health system maturity and funding models, and the presence and nature of the domestic pharmaceutical industry [21] (Table 2). Key informants articulated different governance priorities and conflict of interest concerns depending on the features of their country’s pharmaceutical industry, market size, and national economic objectives related to the domestic pharmaceutical industry.

**Table 2:** Characteristics of the pharmaceutical industry across WHO SEAR countries

Country	Industry size	Nature of industry	Least developed country status	Member of WTO	Use of TRIPS flexibilities
Bangladesh	Large	Domestic production Generic exports	Y	Y	N/A
Bhutan	Small	Import dependent	Y	N	N/A
DPRK	Small	Domestic production	N	N	N/A
India	Large	Domestic production Generic exports	N	Y	Y
Indonesia	Large	Domestic production Generic exports	N	Y	Y
Maldives	None	Import dependent	N	Y	N/A
Myanmar	Small	Import dependent	Y	Y	N/A
Nepal	Medium	Domestic production Import dependent	Y	Y	N/A
Sri Lanka	Small	Import dependent	N	Y	N/A
Thailand	Large	Domestic production	N	Y	Y
Timor-Leste	None	Import dependent	Y	N	N/A

Information in the table is adapted from WHO “Access to medical products in the South-East Asia Region 2021” report [21]

For some countries in the early stages of establishing their regulatory and health systems, priority issues including promoting access to essential medicines and addressing medicines shortages, challenges which were exacerbated by the country’s reliance on imports and donations of pharmaceuticals. Key informants in these countries explained that issues related to conflicts of interest, while considered important, were secondary to efforts to establish the mission and function of pharmaceutical regulators and to build capacity in terms of human resources and technical expertise.

In other countries, particularly those with significant domestic and export pharmaceutical industries, concerns about conflicts of interest and pharmaceutical industry influence within regulatory processes had prompted national conversations and led to significant policy developments over the past decade. One example was the investigation and report in 2012 by 59th Parliamentary Standing Committee into the activities of the Central Drugs Standard Control Organisation (CDSCO), the agency mandated with drug regulation in India [22]. The Committee noted that the mission of the CDSCO at the time was to “meet the aspirations...demands and requirements of the pharmaceutical industry,” (p. 3) and urged the CDSCO to reformulate their primary mission in “unambiguous terms” that prioritised public health. The Committee also identified serious issues related to alleged pharmaceutical industry influence including the “credibility and utility” of opinions from independent experts. This landmark report thus called for major policy development in the area of conflict of interest including instating requirements for mandatory declaration of interests for committee members, creating transparent and written guidelines around the selection of external experts and a mandate to diversify the pool, and emphasizing the provision of evidence in expert opinions [22].

Economic development goals and pressure from international bodies have also prompted policy development around conflicts of interest. For example, Sri Lanka signed onto the United Nations Convention Against Corruption in 2004; the European Commission and International Monetary Fund emphasized Sri Lanka’s obligations under this Convention, prompting creation of a National Action Plan to Combat Corruption and Bribery, an amendment to the Constitution [23], and an array of preventative measures and educational outreach, including a handbook on conflict of interest [24].

Across sampled countries, however, key informants emphasized the need for political leadership and capacity building in preventing and addressing conflicts of interest for committee membership. Key informants agreed on the importance of strong pharmaceutical governance to protect reliable access to affordable, safe, and quality-assured medicines and identified the presence of specific legislation, the existence of oversight bodies, an autonomous regulator, and strong civil society involvement in policy development as key facilitators for developing and implementing conflict of interest policy. However, experts also articulated priority concerns related to understaffing, lack of technical expertise, and lack of trust in emergent regulatory systems, which were key contextual challenges for countries in developing and implementing conflict of interest policy.

### Policies and practices for addressing conflicts of interest

Table 3 summarizes the evidence for disclosure and management of conflicts of interest among public sector pharmaceutical committees in the WHO SEAR. Because many policies are not publicly available or are not available in English, we asked key informants to report policies or practices that are not documented in the public domain and also included accounts of conflict of interest policy development and implementation in the scholarly and grey literature. Eight policy documents (from Bangladesh, Bhutan, India, Nepal, and Sri Lanka) specifically referenced provisions related to conflict of interest. The eight policy documents included: 3 public procurement rules and regulations [25–27]; 2 medicines rules and regulations [28, 29]; 1 medicines regulatory act [30]; 1 national medicines policy [31]; and 1 committee constitution [32].

**Table 3:** Conflict of interest policies and practices in key public pharmaceutical committees in SEAR countries

	Regulation	Selection	Pricing	Public procurement
**Bangladesh**	Quality Policy exists [33]	No information [34]	No information	Procurement Rules and Code of Ethics exists [25] Individual written COI declaration Collective declaration of impartiality Replacement of members with COI Prohibition of gifts, payments, hospitality to members Debarment for violation of COI rules
**Bhutan**	Defines COI [28, 29] Individual written COI declaration prior to all meetings [29] Members with COI must abstain from relevant work [29] Members avail themselves of remuneration for attending meetings [29] *Oversight of policy implementation by Anti-Corruption Commission* *Civil servants prohibited from:* *Working in private sector* *Accepting meals from the private sector*	*Individual written COI declaration prior to all meetings* *Replacement or recusal of members with COI* *Oversight of policy implementation by Anti-Corruption Commission* *Civil servants prohibited from:* *Working in private sector* *Accepting meals from the private sector*	*Individual written COI declaration prior to all meetings* *Replacement or recusal of members with COI* *Oversight of policy implementation by Anti-Corruption Commission* *Civil servants prohibited from:* *Working in private sector* *Accepting meals from the private sector*	Procurement Rules and declaration form [26] Individual written COI declaration prior to all meetings Replacement or recusal of members with COI Disclosure verified by procuring agency Members sign Integrity Pact [35] Prohibition of bribes, consideration, gifts, rewards, favours, or any other material or immaterial benefit or advantage Tender process is open and transparent Grievances mechanism *Oversight of policy implementation by Anti-Corruption Commission* *Civil servants prohibited from:* *Working in private sector* *Accepting meals from the private sector*
**DPRK**	No information	No information	No information	No information
**India**	Individual written COI declaration [22] Subject experts selected from expert bank [36]	Individual written COI declaration [31, 32]	No information [37] *Individual written COI declaration*	N/A – largely at the state level
**Indonesia**	*Individual written COI declaration on annual basis* *Individuals with COI are recused from drug evaluation and committee meetings*	Transparent, evidence-based process for medicines selection [38] *Individual written COI declaration* *Pharmaceutical industry cannot submit formulary requests directly*	*Excludes pharmaceutical industry employees and representatives* *Individual written COI declaration*	No information
**Maldives**	Transparency guideline exists [39] *Individual written COI declaration* *Members with COI must abstain from relevant work* *Agreement to abstain from private sector employment* *Civil servants are provided a non-practice*	No information [40, 41]	N/A – responsibility of regulatory staff	No information
**Myanmar**	No information	No information [42]	No information	No information
**Nepal**	*Civil servants not allowed to work in private sector*	*Civil servants not allowed to work in private sector*	*Industry representatives must declare COI and abstain from relevant work*	Public procurement Code of Conduct [43] Bidders and consultants must provide written COI declaration Prohibits inducements to procurement officials Officials must refrain from COI Officials cannot work for an entity with which they have had procurement dealings until 2 years after retirement Recusal of officials from procurement proceedings where family members are involved Public Procurement Monitoring Office provides oversight and enforcement
**Sri Lanka**	Code of Conduct present Terms of reference exist [30] COI defined Individual written COI disclosure upon selection and from ‘time to time’ thereafter Disclosures recorded in committee minutes Committee members ‘should not’ have any COI Members with COI must abstain from relevant decision-making or other work No employment by pharmaceutical industry 3 years prior or 3 years following membership Subject matter experts do not have voting rights	None	*Individual written COI disclosure required*	*Individual written COI disclosure required*
**Thailand**	Individual written COI disclosure required [44] *Disclosures verified by secretariat*	*Codes of ethics for medicines selection processes* *Excludes pharmaceutical industry employees, owners and executives* Individual written COI disclosure required for NLEM working group experts, [45] *secretariat and NLEM subcommittee during committee development and at each meeting* *NLEM members with any business interest in the medicine or intervention must abstain from relevant decision-making* HITAP members must annually declare COI [46] HITAP members prohibited from receiving financial benefits from private, for-profit companies based on the organisation’s codes of conduct [46] *COI policy is regularly reviewed and revised as needed*	Individual written COI disclosure required [15]	N/A – procurement largely de-centralised
**Timor-Leste**	No information [47]	Transparent decision-making processes [48, 49]	No information	*Anti-corruption commission provides oversight*

Information in this table is based on a review of policies that are publicly available in English, review of secondary literature sources and analysis of key informant interviews (in italics)

In addition, many of the other sampled policies discussed committee governance, ethics, integrity, and underlying values more generally. For example, Nepal’s Public Procurement Act of 2006 and Public Procurement Regulations of 2007 aimed to make public procurement more “open, transparent, and credible,” to “promote competition, clarity, integrity, accountability, and credibility.” Sampled policies also frequently cited other committee documents that suggested that conflict of interest and other governance frameworks might exist but are not publicly available in English. For example, the legislation constituting technical advisory committees frequently specified that the committees, subject to government approval may create their own bylaws, regulate their own procedure, and the conduct of all business to be transacted by it, including establishing expert sub-committees as necessary [50–53]. It is likely that most of these committees have terms of reference and in the event that these or other documents do not already address conflicts of interest, provisions could be added.

#### Defining conflict of interest

Very few policies explicitly defined conflict of interest (*n* = 6/45); others merely referenced conflict of interest disclosure requirements, leaving the definition assumed and open to interpretation. Formal definitions generally encompassed 2 main types of secondary interests that posed a conflict of interest for committee members: 1) the presence of employment, business, and other financial interests in entities with a commercial interest in the decision-making process; and 2) the presence of employment, business, and other financial interests of close relations. Key informants stressed the need to develop shared understandings of what constitutes a conflict of interest within a particular decision-making process and for a particular role, noting that existing policy documents were too generic to provide practical guidance.

Formal definitions identified in policy documents generally only implicitly defined the *primary interest* or obligation that should be given ethical priority. When identified, the primary obligation was not defined in relation to the specific committee, but included “official duties,” “functions,” or “objectivity and independence” in relation to decision-making. The exception were committees engaged in public procurement, which specified the obligations and values that should be given priority in all decision-making such as “economy, efficiency, transparency, fairness and equal treatment of tenders or proposals” [25].

#### Conflict of interest

Overall, we found evidence that committees across pharmaceutical processes consistently required committee members to declare relevant secondary interests (Table 3). Table 4 outlines illustrative types of interests covered by the disclosure requirements. By clearly identifying the secondary interests that required disclosure, committees were implicitly defining what constituted a conflict of interest, even when the policy did not provide a formal definition. Bhutan made their conflict of interest declaration forms publicly available through a website. The forms outlined the types of information committee members were required to disclose. However, across the sample, policies and informants less frequently specified when and how often declarations should occur. One exception was the provisions contained in the Sri Lankan National Medicines Regulatory Act (No. 5 of 2015) requiring that the Minister ensure *prior to appointment* and *periodically*, that prospective members do not have “financial or other conflict of interest in the affairs of the Authority” [30]. The Act specifies that members must disclose the nature of any direct or indirect interest relevant to committee business and that these disclosures be recorded in the committee minutes [30].

**Table 4:** Types of interests covered under current publicly available disclosure requirements

Category of interest	Relevant interests	Illustrative committee examples
**Employment**	Recent, continuing, or planned pharmaceutical industry employment Employment with advocacy organization	“Employment or other professional relationship with any entity directly involved in the production, manufacture, distribution or sale of medicinal products, or directly representing the interest of any such entity in the past 5 years.” [29] (Blood Technical Advisory Committee, Bhutan) “Any employment in a company or organization that may have relevance to the jurisdiction of NMRA including membership of advisory board in the last 5 years or likely to be forthcoming.” (National Medicines Regulatory Authority, National Advisory Committee, Medicines Evaluation Committee, Sri Lanka) “Employment.. . in an entity involved in procurement dealings” [27] (Tender Evaluation Committees, Nepal)
**Financial relationships**	Business dealings Consultancy Paid speaker Paid expert	“Dealings with any company or undertaking which engages in manufacturing, importation, distribution or sale of medicines” [30] (National Medicines Regulatory Authority, National Advisory Committee, Medicines Evaluation Committee, Sri Lanka) “Paid employment including consultancy, commission, paid speaker, paid expert advisor over the past 5 years or likely to be forthcoming” (National Medicines Regulatory Authority, National Advisory Committee, Medicines Evaluation Committee, Sri Lanka)
**Ownership or investment**	Ownership Shares or stocks Self-managed superannuation (pension) fund Partnerships	“Direct, indirect. .. interest in any of the parties participating in the bidding” [35] (Tender Evaluation Committees, Bhutan) “Any other direct or indirect financial interest, example other investments, partnerships plus ownership or a patent for a therapeutic good ownership by employer, investments in self-managed superannuation fund over the past 5 years, or likely to be forthcoming” (National Medicines Regulatory Authority, National Advisory Committee, Medicines Evaluation Committee, Sri Lanka) “Personal or business interests” [25] (Tender Evaluation Committees, Bangladesh) “Encouragement to engage in trade or employment in an area over which the public servant has jurisdiction; encouragement to construct, buy or sell property or speculate in investments by someone involved in procurement” [25] (Tender Evaluation Committees, Bangladesh) “Financial interest (personal or familial) in an entity involved in procurement dealings” [27] (Tender Evaluation Committees, Nepal)
**Board membership**	Company board membership Advisory board membership	“Shareholdings, executive or non-executive board memberships over the past 5 years or likely to be forthcoming” (National Medicines Regulatory Authority, National Advisory Committee, Medicines Evaluation Committee, Sri Lanka)
**Education**	Fellowship Research or education grants Student support	“Fellowship, research or education grants over the past 5 years, or likely to be forthcoming” (National Medicines Regulatory Authority, National Advisory Committee, Medicines Evaluation Committee, Sri Lanka) “Provision by such a company organization of ad hoc support for a patient or student in the last 5 years are likely to be forthcoming.” (National Medicines Regulatory Authority, National Advisory Committee, Medicines Evaluation Committee, Sri Lanka)
**Travel**	Paid travel (flights, train, hotel) Conference registration Invitations to travel or attend training abroad	“Travel plan or conference fee.. .greater than USD $100 over the past 5 years or likely to be forthcoming” (National Medicines Regulatory Authority, National Advisory Committee, Medicines Evaluation Committee, Sri Lanka) “Invitations to visit a foreign country or train abroad” [25] (Tender Evaluation Committees, Bangladesh)
**Gifts**	Monetary gifts or rewards Hospitality Favours or consideration Meetings or entertainment in honour of the public servant Offers of foreign awards, titles, or honours	“Any bribe, consideration, gift, reward, favor or any material or immaterial benefit or any other advantage” [35] (Tender Evaluation Committees, Bhutan) “Hospitality greater than USD $100 over the past 5 years or likely to be forthcoming” (National Medicines Regulatory Authority, National Advisory Committee, Medicines Evaluation Committee, Sri Lanka) “Gifts (except those of small intrinsic value); hospitality, meetings or entertainment to honour or praise the public servant; offers of foreign awards, titles or decorations” [25] (Tender Evaluation Committees, Bangladesh)
**Family interests**	Family or friend ownership or investment	“Private interests. Family’s interests” [2] (Drug Technical Advisory Committee, Bhutan) “Familial interest in any of the parties participating in the bidding” [35] (Tender Evaluation Committees, Bhutan) “A friend or a relation or a financial investment in a business involved in the public procurement transaction” [25] (Tender Evaluation Committees, Bangladesh)
**Clinical trial involvement**	Receipt of research funding or grants Principal investigator on a trial under consideration	“Participation in clinical trials with as principal investigator, contributor of patient or otherwise, involvement as a researcher or in any other capacity in relation to therapeutic goods, or their development in the last 5 years are likely to be forthcoming” (National Medicines Regulatory Authority, National Advisory Committee, Medicines Evaluation Committee, Sri Lanka)
**Intellectual property**	Patents Royalties	“Ownership or a patent for a therapeutic good owned by employer” (National Medicines Regulatory Authority, National Advisory Committee, Medicines Evaluation Committee, Sri Lanka)
**Other**	Anything else	“Please list any of the interests of the kind, such that if you were to be appointed as a member of the NMRA, a description conflict might arise in relation to matters that could come before the authority” (National Medicines Regulatory Authority, National Advisory Committee, Medicines Evaluation Committee, Sri Lanka)

Key informants described a few instances where processes for verifying the accuracy and completeness of conflict of interest disclosure existed. In these cases, members of the secretariat conducted internet searches to verify the individual’s disclosures to the greatest extent possible. In the procurement context, another strategy was to maintain a database of the relationships and business interests of the civil servants and their family members [26]. We found no written information about whether, how, or by whom declarations of interest were evaluated to determine whether a conflict of interest existed or its severity or impact.

#### Prevention and management

To prevent conflicts of interest and mitigate corruption risks, some policies explicitly prohibited certain types of relationships deemed too high-risk. For example, the Bangladesh Public Procurement Rules (2008) prohibit the offer of gifts, hospitality, honours, offers of foreign travel or reward, and encouragement to engage in trade, employment, or other transactions to those involved in the procurement process [25]. The Sri Lankan National Medicines Regulation Authority Act also requires that the Minister ensures that prospective members do not have “financial or other conflict of interest in the affairs of the Authority” prior to appointment and periodically; individuals are disqualified for committee membership if they have been employed by the pharmaceutical industry in the 3 years prior to their committee appointment [30].

Key informants also described standard operating procedures, policy provisions, and practices that were designed to prevent conflicts of interest from occurring in the first instance or altering the situation to eliminate or mitigate the impact of a conflict of interest. These documents were not publicly available in English. Strategies for removing or minimizing the impact of secondary interests deemed at high risk of compromising an individual’s primary obligation included reorganizing roles and responsibilities, or requiring shared decision making and additional checks and balances.

When discussing experiences with conflicts of interest, key informants largely described conflicts of interest arising from part-time employment in the private health sector, such as pharmacies, clinics, or laboratories, and family and friends’ employment in or ownership of private health-related entities. They less frequently raised the issue of committee members’ relationships with the pharmaceutical industry, with many remarking on the small pharmaceutical industry presence in their countries. Consequently, most reported that their institution’s conflict of interest policy primarily focused on prohibiting public sector employees from ‘moonlighting’ in the private sector or requiring recusal from pharmaceutical processes that affected the interests of close relations to avoid the risk of favouritism or nepotism. They explained that in the presence of such conflicts of interest, “there are favours which [civil servants] can do,” because “they know well how the system here works,” thus creating a risk of preferential treatment or at the extreme, fraud.

We found little information in sampled policies regarding management of conflicts of interest. Table 5 outlines the specific strategies identified including recusal from deliberations, meetings, or procurement proceedings as the key management strategy. We did not identify any details regarding who should evaluate whether a conflict of interest existed, the severity of the conflict, and whether and how it should be managed. Key informants identified a need for clear guidance around how to manage conflicts of interest consistently, proportionately, and transparently.

**Table 5:** Conflict of interest management strategies identified in publicly available policies

Management strategy	Examples
Recusal from activities, meeting or relevant work in which the individual has an interest	Bhutan Blood Technical Advisory Committee [30] Nepal Bid and Tender Evaluation Committees [28] Sri Lanka National Medicines Regulatory Authority, National Advisory Committee, and the Medicines Evaluation Committee [31] Thailand HITAP [47]
Replacement of committee members with conflict of interest for specific tenders	Bangladesh Tender Committees [26] Bhutan Tender Committees [27]
Subject matter experts able to provide testimony but unable to vote	Sri Lanka National Medicines Regulatory Authority, National Advisory Committee, and the Medicines Evaluation Committee [31]

#### Transparency and oversight

We did not find any instances of public transparency around conflict of interest disclosures of committee members: that is, we did not find that conflict of interest disclosures were published or otherwise made available for public scrutiny, in full or summary form. For example, none of the published Essential Medicines Lists, while listing the committee membership, included the members’ conflict of interest disclosures.

A few of the sampled countries had anti-corruption commissions which provided guidance or oversight of conflict of interest policy implementation. In cases where the anti-corruption committee performed regular audits, key informants described high compliance with maintaining written declaration of interests (but not public disclosure). In other cases, key informants perceived that the anti-corruption commission did not have the political independence or human resources to carry out this function effectively.

### Creating conditions to effectively address conflicts of interest

Key informants emphasized the importance of regulatory autonomy, independence, and strength to create the conditions in which conflicts of interest can be identified and decisively managed. Ultimately, they believed regulatory autonomy from the government, which might be the main pharmaceutical importer or responsible for facilities, and the private sector (e.g., through rejection of user fees as a funding mechanism) would build trustworthiness and make the regulator effective in fulfilling its mission. One key informant expressed,

I think if more needs to be done, it needs to be the watchdogs that need to be stringent...there has to be a point where an honest institution, which is not really a paper tiger can really look into what nefarious stuff is going on. But yes, that’s a difficult task.

Key informants characterized regulatory autonomy and independence both financially and functionally. The presence of an independent oversight body that was adequately resourced seemed to be a motivator and facilitator for public sector pharmaceutical committees to develop and implement conflict of interest provisions. For example, key informants and the literature identified Bhutan’s Anti-Corruption Commission as a model in the WHO SEAR. Transparency International conducted an independent assessment of Bhutan’s Anti-Corruption Commission and found that despite challenges related to limited human and other resources, the Commission had a clear mandate and vision, well-established capacities, and had made strong contributions to investigation, research, outreach, education, and prevention, including addressing conflicts of interest [54].

In-country experts on conflicts of interest emphasised the need for greater transparency around disclosure and management of conflicts of interest. They emphasised that first, disclosures are necessary and should be made in the context of a shared, public understanding of what constitutes a conflict of interest. Second, policy around how conflicts will be managed should be publicly available and should specify which conflicts of interest will preclude participation. Third, all decisions taken should be publicly reported so that the public can compare these actions to the intended policy, assess compliance, and hold committees accountable. Finally, a redressal mechanism should be put in place to handle policy violations.

Key informants pointed to the important roles of civil society actors in prompting conflict of interest policy developments by calling for greater transparency and holding policymakers accountable. One informant explained that in response to civil society advocacy, regulatory agencies had divested from industry sponsorship or withdrawn from industry-funded programs or events: “This was the behavioural change that has happened from past couple of years, because now that we have very strong civil society voice, they just can’t have these kinds of notorious partnerships as well.” A supportive legislative framework also provided civil society with mechanisms to bring transparency to policy processes. Key informants identified Freedom of Information laws, anti-corruption legislation, and system-wide regulation as effective mechanisms for creating awareness of conflicts of interest and preventing or minimising particular conflicts of interest for civil servants related to gift-giving and employment or other financial relationships with the private sector.

Thailand’s Health Intervention and Technology Assessment Program (HITAP) program provides a model for a principled, consistent, and transparent approach to conflict of interest management, with emphasis on conflict avoidance [46]. In 2012, Thailand’s HITAP developed seven principles of good governance: transparency, inclusiveness, accountability, quality, timeliness, consistency, and contestability to guide its work [46]. Consistent with these principles, HITAP has a strict conflict of interest policy for researchers and for the Programme as a whole, laid out in a personal and institutional code of conduct. Both individual researchers and the Programme are prohibited from receiving benefits such as research grants, sponsorship to attend conferences and training courses, or other direct and indirect benefits from private, for-profit companies [46]. Staff must annually complete a disclosure of all interests, and depending on the nature of conflicts of interest disclosed, individuals may be precluded from undertaking particular types of work [46]. Transparency and accountability are emphasised as “key” to maintaining HITAP’s independence, public trust, and the perceived credibility of work products:

Anticipating and learning how to handle commercial attempts to acquire improper influence, many of which are far subtler than banknotes proffered in brown paper envelopes, will need to form a part of everyone’s training. It will not be enough (indeed, it is already not enough) to be scrupulously honest. HITAP has to be seen to be scrupulously honest. Transparency and accountability will be the keys to maintaining HITAP’s future independence [46] (p. 196).

As a consequence of HITAP’s principles, the potential of HITAP’s work to support decision-making authority could grow, necessitating even greater emphasis on independence, transparency, and avoidance of conflicts of interest.

## Discussion

Countries within the WHO SEAR are highly diverse in terms of size, health system maturity and funding models, and the nature of the pharmaceutical industry. The WHO SEAR thus provides a useful case study for understanding the nature and import of conflict of interest within public sector pharmaceutical processes. Countries pursuing national goals related to Universal Health Coverage or increased access to safe, affordable, quality essential medicines have identified governance as a strategic priority in their National Medicines Plans [55]. Though countries grappled with priority public health, human resource, and regulatory concerns, many national pharmaceutical committees have policies and practices to identify and manage conflicts of interest, such as disclosure requirements and processes to recuse individuals with conflicts of interest from relevant work. Some, like Thailand’s HITAP committee, are internationally recognized for their practices [46]. Yet gaps remain around how to prevent, manage, and enforce policies on conflicts of interest. These gaps are not unique to the countries studied, but are reflected in the conflict of interest policies of many pharmaceutical and health-related committees globally [9]. For example, across health-related organizations, there is an over-reliance on disclosure as a management strategy, an emphasis on management of conflicts of interest instead of prevention, and a lack of transparency and accountability around policy implementation and enforcement [9].

Conflicts of interest are highly context-specific, depending on the institutional setting, the individual’s roles and responsibilities, and their primary obligation. In this study, key informants identified the need for greater education and specificity around what constitutes a conflict of interest in a given context. Very few policies explicitly defined a conflict of interest; others merely referenced conflict of interest or declaration requirements, leaving the definition assumed. This is consistent with an analysis of conflict of interest policy among prominent guideline developers, HTA, and scientific advisory committees in the United States, United Kingdom, Canada, and Australia, which found that only 59% (13/22) explicitly defined the primary interest or obligation at stake [9]. In contrast, all 22 sampled policies (100%) clearly defined and specified the types of secondary interests that required disclosure [9]. If the primary interest is not explicit, it is challenging to determine the relevance of disclosed interests [56] or to evaluate the existence or severity of a conflict of interest. Specifically, to strengthen disclosure processes, it is necessary to clearly define the *primary* interest or obligation as well as what constitutes a *secondary* interest so that the existence and severity of a conflict of interest can be clearly, transparently, and objectively evaluated, allowing for the prevention or management of conflict of interest.

In the current study there was overall recognition of the importance of disclosure as a means to identify committee members’ interests. In some cases, disclosure processes were clearly operationalized, typically in the form of a written declaration of interests that committee members were required to complete at the outset of the committee’s activities. More often, however, there was little detail as to how, when, and how often disclosure should or would occur. Furthermore, authors of sampled policies and key informants often conceptualized disclosure as one of the main management tools. However, while disclosure is necessary, it is not sufficient for managing conflicts of interest [8, 57]. In the absence of verification, evaluation, and management, disclosure becomes merely a bureaucratic exercise. Disclosure should be seen as a means to an end and should encompass a culture of openness, a process for disclosure, and specific instruments for documentation purposes.

Preventive approaches may be the most effective and least resource-intensive strategies for assuring the independence of decision-making within public sector pharmaceutical committees. A preventive approach entails a very clear understanding of the nature of conflict of interest within a particular process, and the kinds of conflicts that pose the greatest risk to the committee’s primary interest. The WHO has identified the development of clear, consistent selection criteria for members of pharmaceutical public sector committees as a priority strategy [10], which could include rules around selecting committee members who are free or willing to divest from conflicting commitments deemed high risk.

There are many challenges associated with enacting these stricter selection criteria. Widespread institutional and/or broader societal acceptance of private sector involvement in public sector and health-related decision-making processes can have significant detrimental impacts on the development, implementation, and enforcement of conflict of interest policies. As in many high-income countries, many physicians and researchers rely on pharmaceutical industry funding for continuing medical education or research. The shortage of independent experts to fill committee roles may be particularly felt in countries with a small population and a limited number of health professionals and technical experts. Globally, there is a lack of policy to govern physicians’ and researchers’ interactions with industry in medical education, universities, and in practice [4], and public transparency remains the dominant approach [8, 58]. Pervasive pharmaceutical promotion and industry influence over prescribing and dispensing habits as well as continuing medical and pharmacy education will likely be a key implementation challenge for countries’ pharmaceutical committees. Furthermore, in countries with a strong local pharmaceutical manufacturing industry, there may be competing political priorities such that strong conflict of interest policies are difficult to introduce and enforce.

In order to better develop clear processes and procedures for conflict of interest disclosure, prevention, and management, building institutional cultures of transparency, accountability, and trust may be a requisite first step. Our findings suggest that these cultures thrive within strong, independent institutional contexts, bolstered by clear guiding principles and a supportive legislative framework. Conversely, for public institutions, partnering with industry may create a means for commercial interests to shift policy agendas, influence the process of problem identification and root cause diagnosis, and propose solutions that are favourable to industry interests, but diverge from the core functions, obligations and mission of the public institution [7]. Ultimately, this can also undermine the trust and confidence the public has in policymakers, the policy process, and its outcomes [7]. Thus, while conflict of interest policy can address the relationships between individuals and industry, it is one, but not the only tool for addressing industry influence within public health initiatives and public sector decision-making more broadly [59]. For example, researchers have proposed a tool for assessing national governance of public health initiatives led by multinational corporations, which clearly outlines expectations regarding regulation, information sharing, stakeholder engagement, and accountability, among others [60].

Taking these understandings into account, we identify the following priorities among policy approaches to conflict of interest:

- Identifying the appropriate institutional bodies to create, implement and enforce conflict of interest policy and practices related to public sector pharmaceutical committees and ensuring their independence and sustainability;
- Developing context-specific education and guidance about what constitutes a conflict of interest within a particular decision-making process (including clear definitions of the primary and secondary interests);
- Promoting consistency in disclosure processes across committees and members, and ensuring that disclosure happens on an ongoing basis;
- Developing practical strategies to prevent, evaluate, and mitigate the conflict of interest;
- Mechanisms to make decisions and data related to conflicts of interest publicly available to promote accountability.

### Limitations

Our study is limited by its reliance on publicly-available, English language documents as a primary data source. There were a number of countries whose key policy documents are in languages other than English (e.g,. Bangla, Thai) or were in formats that could not be read or processed using Google Translate (e.g., a scanned copy of a type-written document). We mitigated this by verifying with key informants whether policies existed and sought additional information about policies during interviews; some informants were able to source additional English language policy documents for us. Yet, it is likely that information and perspectives were missed and because key informant interviews were used as a supplementary data source, we did not reach saturation in terms of the range of perspectives or depth of analysis. The scope of the study was limited to examining conflict of interest management policies by national-level committees and agencies only. However, we recognize that in some of the countries, key aspects of the pharmaceutical process are decentralized such a state-level public procurement. Thus, key committees and agencies may function at sub-national levels in accordance with subnational policies and we have not addressed these processes. The implications of decentralized policies and processes addressing conflicts of interest is an important area for future research. The limitations of our approach to sampling and analysing policies were mitigated by the triangulation of key informant interview data and robust sampling of the scholarly and grey literature, much of which has been written by ministry employees, civil servants, and thought leaders in these countries.

## Conclusion

Effectively addressing conflicts of interest requires leadership, clear policies, and well-embedded systems of practice. This policy survey showed that in some countries there is growing awareness around conflicts of interest, but gaps remain related to organised policy and practice to prevent and manage conflicts of interest; others have well-established practices for identifying conflicts of interest, but do not use the information for management. Priority concerns related to understaffing, lack of trust in emergent regulatory systems, and industry influence within medical education and practice may create key contextual challenges for countries in developing and implementing conflict of interest policy. Our study also highlights selected practices within the WHO SEAR that others could draw on to develop more robust and transparent pharmaceutical policies and practices of their own. While clear processes and procedures for conflict of interest disclosure and management are useful, further work should explore upstream approaches to building cultures of transparency, accountability, and trust.

### Supplementary Information

**Additional file 1.**

## Data Availability

All source documents for the policy analysis are publicly available and are cited in Table 2. Interview data not available as we did not obtain consent to share these data and social and privacy risks to participants associated with re-identification are significant.

## Appendix 1

### Reviewing Conflict of Interest Management Practices in the Pharmaceutical Sector in the South-East Asia Region

#### Key informant interview guide

**Preamble:** We are researchers at the University of Toronto and the World Health Organization South-East Asia Region Office interested in understanding how conflicts of interest are identified, disclosed and managed within public sector pharmaceutical committees.

Pharmaceutical systems are technically complex and involve extensive interactions between the private sector and the public sector. Private sector interests can influence what products are selected for reimbursement or procurement, prices of health products, and how health products are used. We are particularly interested in your experience and perspectives on how to manage such interests.

**Table:**

Question	Prompts	Notes
Please tell me a bit about the work that [name of committee] does and your role in relation to this committee.		
In what ways does [name of committee] or committee members interact with the private sector?	Please tell me about a specific time that interaction with the private sector was beneficial. Please tell me about a specific time that interaction with the private sector created challenges.	
Please describe the ways that [name of committee] typically manages the role and influence of the private sector.	Prompt for specific examples of procedures, practices, or processes.	
What works well in terms of procedures and processes related to managing conflicts of interest?		
What are the current challenges or barriers facing [name of committee] in terms of managing conflicts of interest?		
How do you feel conflicts of interest should be managed?		
Is there anything else you would like to share?		

## Appendix 2

### Catalogue of included documents and corresponding pharmaceutical committees

**Table:**

Country	Committee type	Document Type	Document name	Document author	Name of committee	Committee selection information	COI provisions
**Bangladesh**	Market authorization, Licensing, Quality assurance	Pharmaceutical legislation	The Drugs Act, 1940 (ACT NO. XXIII OF 1940)	Government of the People’s Republic of Bangladesh	The Drugs Technical Advisory Board	N	N
		Pharmaceutical legislation	Drug Control Ordinance Act 1982	Government of the People’s Republic of Bangladesh	Drug Control Committee	Y	N
		Committee Terms of Reference or COI policy	Quality Policy	Directorate General of Drug Administration (DGDA)	All DGDA committees	N	N
		Pharmacovigilance guideline	National Guideline for Pharmacovigilance	Directorate General of Drug Administration (DGDA)	Adverse Drug Reaction Advisory Committee	N	N
	Medicine selection	National Medicine Policy	National Drug Policy 2016	Directorate General of Drug Administration (DGDA)	Drug Control Committee	Y	N
	Procurement	Procurement regulation	The Public Procurement Rules 2008	Central Procurement Technical Unit, Implementation Monitoring and Evaluation Division, Ministry of Planning	Tender Committees	Y	Y
**Bhutan**	Market authorization, Licensing, Quality assurance	Pharmaceutical legislation	Medicines Act of the Kingdom of Bhutan, 2003	Kingdom of Bhutan	Drugs Technical Advisory Committee	Y	N
		Pharmaceutical regulation	Bhutan Medicines Rules and Regulations, 2019	Bhutan Medicines Board	Drugs Technical Advisory Committee	Y	Y
		Pharmaceutical regulation	Blood and Blood Products Regulation, 2016	Drug Regulatory Authority	Blood Technical Advisory Committee	Y	Y
	Medicine selection	National Medicine Policy	National Drug Policy 2007	Ministry of Health	Advisory Technical Committees	Y	N
	Procurement	Procurement regulation	Procurement Rules and Regulations 2019	Ministry of Finance, Royal Government of Bhutan	Tender Committees	Y	Y
**India**	Market authorization, Licensing, Quality assurance	Pharmaceutical legislation	Drugs and Cosmetics Act, 1940 and Rules 1945 as amended up to December 2016	Ministry of Health & Family Welfare, Government of India	Drugs Technical Advisory Board; The Drugs Consultative Committee	Y	N
		Committee Terms of Reference or COI policy	By-laws of the DTAB	Ministry of Health & Family Welfare, Government of India	Drugs Technical Advisory Board; The Drugs Consultative Committee	Y	N
		Committee Terms of Reference or COI policy	Proposed List of Subject Experts in Various Therapeutic Areas	Ministry of Health & Family Welfare, Government of India	Subject Expert Committees (SECs)	Y	N
	Pricing and reimbursement	National Medicine Policy	Pharmaceutical Policy 2002	Department of Pharmaceuticals (DoP), Ministry of Chemicals & Fertilizers	National Pharmaceutical Pricing Authority	N	N
	Medicine selection	National Medicine Policy	National Vaccine Policy 2011	Ministry of Health & Family Welfare	National Technical Advisory Group on Immunization (NTAGI)	Y	Y
		Committee Terms of Reference or COI policy	Constitution of Standing National Committee on Medicines (SNCM) for revision of National list of Essential medicines (NLEM)	Ministry of Health & Family Welfare	Core Committee on National List of Essential Medicines	Y	Y
	Pricing and reimbursement	Committee Terms of Reference or COI policy	Function of National Pharmaceutical Pricing Authority	Department of Pharmaceuticals (DoP), Ministry of Chemicals & Fertilizers	National Pharmaceutical Pricing Authority	Y	N
		Pricing Policy	National Pharmaceutical Pricing Policy 2012	National Pharmaceutical Pricing Authority	NELM Expert Core Committee; National Pharmaceutical Pricing Authority	Y	N
**Indonesia**	Market authorization, Licensing, Quality assurance	Committee Terms of Reference or COI policy	Web pages: Background, Duty, Main Functions, Authority; Corporate Culture; Basic Principles; Conceptual Framework; Strong Organization	National Agency of Drug and Food Control (NA-DFC) (Badan Pengawas Obat dan Makanan, Badan POM)	National Committee on Drug Evaluation	N	N
	Medicine selection	National Medicine Policy	National Medicine Policy 2006	Ministry of Health	National Essential Medicines Selection Committee	Y	N
		Committee Terms of Reference or COI policy	Health Technology Assessment (HTA) Guideline	Ministry of Health	Indonesian Health Technology Assessment Committee (InaHTAC)	N	N
**Maldives**	Market authorization, Licensing, Quality assurance	National Medicine Policy	National Medicine Policy – 2007	Maldives Food and Drug Authority (MFDA)	Medicines Board	N	N
		National Medicine Policy	National Medicine Policy – 2018-2023	Maldives Food and Drug Authority (MFDA)	National Pharmaceutical Board	Y	N
		National Medicine Policy	National Blood Policy 2018	Ministry of Health	National Blood Council	N	N
	Medicine selection	Essential Medicines List	Essential Medicines List – 2018	Maldives Food and Drug Authority, Medicine and Therapeutic Goods Division	Essential Medicines List core committee	Y	N
**Myanmar**	Market authorization, Licensing, Quality assurance	Pharmaceutical legislation	National Drug Law 1992, Amendment to National Drug Law 2014	Republic of the Union of Myanmar	Food and Drug Board of Authority	Y	N
		National Medicine Policy	National Medicines Policy – 2019	Ministry of Health and Sports, Department of Food and Drug Administration (DFDA)	Technical working groups	Y	N
	Medicine selection	Essential Medicines List	National List of Essential Medicines – 2016	Essential Drug Program, Medical Care Division, Department of Medical Services	NLEM Task Force Committee	Y	N
**Nepal**	Market authorization, Licensing, Quality assurance	Pharmaceutical legislation	Drugs Act 1978	Government of Nepal	Drugs Advisory Council and Drugs Advisory Committee	Y	N
		Committee Terms of Reference or COI policy	Drugs Advisory Council and Drugs Advisory Committee Formation Rules, 2037(1970)	Government of Nepal	Drugs Advisory Council and Drugs Advisory Committee	Y	N
		Committee Terms of Reference or COI policy	Web page Mission & Vision and Role of DDA	Department of Drug Administration, Ministry of Health and Population	Drug Consultative Council; Drug Advisory Committee	N	N
		National Medicine Policy	National Drug Policy 1995 (draft 2007)	Department of Drug Administration, Ministry of Health and Population	Drug Advisory Committee	N	N
	Medicine selection	Essential Medicines List	National List of Essential Medicines – 2016	Department of Drug Administration, Ministry of Health and Population	NELM Main and Draft Committees	Y	N
	Procurement	Procurement regulation	The Public Procurement Act (PPA), 2007 (2063)	Government of Nepal	Tender evaluation committees	Y	Y
**Sri Lanka**	Market authorization, Licensing, Quality assurance	Pharmaceutical legislation	National Medicines Regulatory Authority Act, 2015	Democratic Socialist Republic of Sri Lanka	National Medicines Regulatory Authority	Y	Y
		Pharmaceutical legislation	Cosmetics, Devices and Drugs Act (CDDA) No. 27 of 1980 with several amendments from 1985	Democratic Socialist Republic of Sri Lanka	Cosmetics, Devices and Drugs Technical Advisory Committee	Y	N
		National Medicine Policy	National Medicine Policy 2005	Ministry of Health, Nutrition and Indigenous Medicine	Essential Medicine committee	N	N
**Thailand**	Market authorization, Licensing, Quality assurance	Pharmaceutical legislation	Drugs Act BE 2510 (1967), amended last in 1987	Government of Thailand	Drug Committee	Y	N
		Committee Terms of Reference or COI policy	Web page Mission & Vision and Roles and Responsibilities	Thai Food and Drug Administration (Thai FDA)	National Drug Committee	N	N
	Medicine selection	Committee Terms of Reference or COI policy	Web pages	Ministry of Public Health	Health Intervention and Technology Assessment Programme (HITAP)	N	N
**Timor-Leste**	Market authorization, Licensing, Quality assurance	Pharmaceutical legislation	Decree Law No 12/2004	Democratic Republic of Timor-Leste Government	Commission for the Selection of Medicines, Medical Products and Medical Equipment	Y	N
		National Medicine Policy	National Drug and Medicines Policy 2010	Ministry of Health Republic of Timor-Leste	Advisory Board	N	N
		National Medicine Policy	National Drug and Medicines Policy 2017	Ministry of Health Republic of Timor-Leste	National Medicines and Therapeutic Committee	N	N
	Medicine selection	Essential Medicines List	Timor-Leste Essential Medicines List-2015	Committee for selection of medicines, products and medical equipment with Department of Pharmacy, Ministry of Health	EML Committee	Y	N

## Abbreviations

CDSCO: Central Drugs Standard Control Organisation
CDER: United States Food and Drug Administration Center for Drug Evaluation and Research
HITAP: Health Intervention and Technology Assessment Program
ICH: International Council for Harmonisation of Technical Requirements for Pharmaceuticals for Human Use
NGO: Non-Governmental Organization
SEAR: South-East Asia Region (Bangladesh, Bhutan, India, Indonesia, Maldives, Myanmar, Nepal, Sri Lanka, Thailand, and Timor-Leste)
WHO: World Health Organization
